# Supplementary material for: Evaluating the Impact on Clinical Task Efficiency of a Natural Language Processing Algorithm for Searching Medical Documents: Prospective Crossover Study
Source: JMIR Med Inform. 2022 Oct 26;10(10):e39616. doi: 10.2196/39616 (PMC9647457; doi:10.2196/39616)
Supplement: Multimedia Appendix 1 [file medinform_v10i10e39616_app1.pdf]

Evaluator Review Questionnaire

Please only complete this once you have finished all 3 tasks. Any feedback is helpful so please be as thorough and as honest as you can when completing this questionnaire.

The survey will take ~ 10 minutes to complete and all responses are anonymous.

Section 1 ...

Background Information

The data analysts will NOT be linking responses to named individuals

1. Please type in (or copy & paste) your patient-centric viewer username (e.g. eval00az). *

Enter your answer

2. How many years of clinical experience do you have? *

- ○ Pre-clinical or intercalating medical student
- ○ Clinical years medical student
- ○ 1-5 years of clinical experience
- ○ 6-10 years of clinical experience
- ○ 11+ years of clinical experience

3. For those doctors working clinically (or are recently retired), what specialty do/did you work in day-to-day? (Please enter N/A if irrelevant) *

Enter your answer

4. Please list the types of medical record systems you use regularly to search through EHRs. *

Enter your answer

5. What search functionality are you accustomed to using in your local EHRs? *

- ☐ No search
- ☐ String search
- ☐ Other

6. If you answered "Other" to the above question, please expand:

Enter your answer

7. Did you use the training patient (David Lee) before starting the tasks to familiarise yourself with the patient-centric viewer and search functionalities? If yes, how useful was the training patient? *

- ○ Very useful
- ○ Somewhat useful
- ○ Somewhat not useful
- ○ Not very useful
- ○ I didn't use the training patient

Section 2 ...

Patient Documents and Clinical Scenarios

This section will ask about the quality of the synthesised patients and clinical scenarios, as well as relevance of the questions.

8. With regards to the information you were provided with during the tasks, how realistic did you find the following: *

	Very unrealistic	Somewhat unrealistic	Somewhat realistic	Very realistic	Not enough clinical experience to say
The patient documents/notes in the patient-centric viewer	○	○	○	○	○
The clinical scenarios described in the Evaluation Platform (e.g. "The patient is complaining of musculoskeletal pain and is asking for pain relief...")	○	○	○	○	○

9. How strongly do you agree/disagree with the following statements: *

	Strongly disagree	Somewhat disagree	Somewhat agree	Strongly agree	Not enough clinical experience to say
The questions asked were relevant to the clinical scenarios provided.	○	○	○	○	○
The questions prompted me to find information I would want to find out about a patient during their care.	○	○	○	○	○
The questions tested search functionality more than medical knowledge or clinical judgement.	○	○	○	○	○
The questions and scenarios were easy to understand.	○	○	○	○	○
I had sufficient medical knowledge to answer all of the questions.	○	○	○	○	○
The patients' conditions were broad and encompassed a range of specialities.	○	○	○	○	○

10. Any further comments about the patient scenarios, tasks or questions?

Enter your answer

Section 3 ...

Search functionalities

There is commonly no search functionality within Electronic Health Records (EHR) in the UK. This section will ask about your experience using different search functionalities within a simulated EHR system (the patient-centric viewer).

11. How strongly do you agree/disagree with the following statements (please read them carefully!): *

	Strongly disagree	Somewhat disagree	Neither agree or disagree	Somewhat agree	Strongly agree
String searching enabled me to find relevant information FASTER than having no search function.	○	○	○	○	○
The NLP-enhanced search algorithm enabled me to find relevant information FASTER than having no search function.	○	○	○	○	○
The NLP-enhanced search algorithm enabled me to find relevant information FASTER than string searching.	○	○	○	○	○
String searching enabled me to find MORE RELEVANT information than having no search function.	○	○	○	○	○
The NLP-enhanced search algorithm enabled me to find MORE RELEVANT information than no search function.	○	○	○	○	○
The NLP-enhanced search algorithm enabled me to find MORE RELEVANT information than string searching.	○	○	○	○	○
Higher search functionality was advantageous for all types of questions.	○	○	○	○	○
Being able to STRING SEARCH patient notes will make clinical workflows more efficient than having NO search.	○	○	○	○	○
Being able to search patient notes using the NLP-ENHANCED SEARCH will make clinical workflows more efficient than having NO search.	○	○	○	○	○
Being able to use NLP-ENHANCED SEARCH for patient notes will make clinical workflows more efficient than using STRING SEARCH.	○	○	○	○	○
I was worried about missing relevant information when string searching.	○	○	○	○	○

12. Any further comments regarding your experience using the different search functionalities?

Enter your answer

Section 4 ...

The NLP-enhanced search tool

The NLP-enhanced search tool is designed to provide a higher search functionality than simple string search by including abbreviations, acronyms and other related terminology of search queries in the highlighted findings.
(e.g. while simple string search would miss a mention of misspelt "codiene", the NLP-enhanced search tool would pick this up as a misspelt version of codeine).

This section will ask about your experience using the NLP-enhanced search tool.

13. How strongly do you agree/disagree with the following statements: *

	Strongly disagree	Somewhat disagree	Somewhat agree	Strongly agree
The NLP-enhanced search algorithm consistently brought back unrelated findings.	○	○	○	○
Results from NLP-enhanced search distracted me from finding the information I was looking for.	○	○	○	○
Unrelated findings significantly impacted the usefulness of the NLP-enhanced search algorithm.	○	○	○	○
NLP-enhanced search was NOT useful for VAGUE search terms such as ‘disease’, ‘medication’ or ‘surgery’.	○	○	○	○
NLP-enhanced search was NOT useful for SPECIFIC search terms such as ‘warfarin’, ‘hypertension’ or ‘HbA1c’.	○	○	○	○
I was worried about missing relevant information when using NLP-enhanced search.	○	○	○	○

14. Were there any findings (highlighted word(s)) from your NLP-enhanced searches that surprised you?

Enter your answer

Section 5 ...

Final comments

Thank you once again for taking part in this clinical evaluation. Your help is much appreciated!

The space below is for any final feedback you would like to provide.

15. Any final comments on your overall experience?

Enter your answer
